# Targeting focal adhesion kinase inhibits cell migration and non-angiogenic vascularization in malignant breast cancer

**DOI:** 10.1007/s12282-025-01792-6

**Published:** 2025-10-28

**Authors:** Misato Masuyama, Masafumi Shimoda, Ikumi Seto, Kaori Kikumori, Kaori Abe, Nanae Masunaga, Chieko Mishima, Masami Tsukabe, Tetsuhiro Yoshinami, Yoshiaki Sota, Tomohiro Miyake, Tomonori Tanei, Kenzo Shimazu

**Affiliations:** Department of Breast and Endocrine Surgery, The University of Osaka Graduate School of Medicine, 2-2-E10, Yamadaoka, Suita, Osaka 565-0871 Japan

**Keywords:** Breast cancer, Defactinib, Drug resistance, Focal adhesion kinase, Neovascularization

## Abstract

**Background:**

High-grade carcinomas, including breast cancer, are associated with angiogenesis and non-angiogenic vascularization. Non-angiogenic vascularization enhances blood flow, tumor growth, and metastasis. Although inhibition of focal adhesion kinase (FAK) is a promising anticancer strategy, its effect on non-angiogenic vascularization remains unknown. We aimed to determine if defactinib, an oral selective FAK inhibitor, suppresses tumor growth by inhibiting non-angiogenic vascularization via reduced cell migration in malignant breast cancer cell lines.

**Methods:**

Non-angiogenic vascularization was evaluated in JIMT-1 and MDA-MB-231 cells. Western blotting, fluorescent immunocytochemistry, cell migration assay, time-lapse microscopy, and tube formation assays were performed to evaluate the effects of defactinib. Cells were transfected with siFAK to evaluate off-target effects. The in vivo effects were assessed in orthotopic mouse tumor models, followed by immunohistochemical staining of excised tumor samples.

**Results:**

Defactinib induced changes in cell morphology, migration, and the formation of vascular-like structures in JIMT-1 and MDA-MB-231 cells. Phosphorylation of FAK/PTK2 and its downstream effectors was reduced in defactinib-treated cells. SiRNA-mediated FAK knockdown produced similar effects. Treatment with defactinib suppressed tumor growth in orthotopic mouse tumor models, resulting in tumor shrinkage and reduced macroscopic blood-rich areas. Immunohistochemical staining also revealed a significant reduction in the number of vessels characterized by human serpin family E member 2—a potential indicator of non-angiogenic vascularization—in the defactinib-treated group, with no significant increase in apoptosis.

**Conclusions:**

FAK inhibition was shown to suppress non-angiogenic vascularization. Defactinib has the potential to serve as a novel treatment for malignant breast cancer which is resistant to conventional therapies.

**Supplementary Information:**

The online version contains supplementary material available at 10.1007/s12282-025-01792-6.

## Introduction

Tumor growth and metastasis require adequate oxygen and nutrients. In addition to tumor angiogenesis, non-angiogenic vascularization is essential for providing the increased oxygen and nutrients required for tumor growth. Bevacizumab, an anti-vascular endothelial growth factor inhibitor, is widely used as a targeted therapy for various cancers. Since 2011, the combination of bevacizumab and paclitaxel has been approved in Japan for the treatment of inoperable or recurrent breast cancer. Bevacizumab reduces tumors and temporarily suppresses metastatic breast cancer progression, improving progression-free survival (PFS) and response rates. However, results from clinical trials suggest that bevacizumab does not extend overall survival [1, 2]. This lack of overall effect may be due to the increased plasma levels of angiogenic- and hypoxia-related inflammatory cytokines in non-responders that induce a more aggressive tumor phenotype [3].

Non-angiogenic vascularization has emerged as a potential therapeutic target in aggressive breast tumors, such as triple-negative breast cancer (TNBC) and tumors resistant to conventional therapies [4]. Two well-characterized forms of non-angiogenic vascularization are vasculogenic mimicry (VM) and vessel co-option (VCO). In VM, cancer cells acquire endothelial-like properties and form vascular-like structures [5], which are associated with rapid tumor growth and metastasis [6, 7], leading to a poor prognosis [8, 9]. We previously demonstrated that salinomycin, which targets breast cancer stem cells (CSCs) [10], inhibits VM activity in human epidermal growth factor receptor 2 (HER2)-positive breast cancer cell lines and clinical samples. Salinomycin alters cancer cell cytoskeleton morphology, reduces cell migration ability, and potentially suppresses VM [11]. However, the toxicity of salinomycin in humans limits its clinical application.

Mechanistically, VM differs from traditional angiogenesis and may be resistant to conventional antiangiogenic therapies [12]. Research into drugs targeting molecular pathways involved in VM formation—such as vascular endothelial cadherin, erythropoietin-producing hepatoma receptor A2, focal adhesion kinase (FAK) encoded by *PTK2*, and matrix metalloproteinases—is ongoing, but these approaches have not yet reached clinical application. The mechanisms underlying VM are not yet fully understood, making the development of effective diagnostic and therapeutic strategies particularly challenging [13]. VCO also contributes to resistance to antiangiogenic therapies [14]. In VCO, cancer cells migrate toward and surround pre-existing blood vessels, incorporating them into the tumor architecture [15]. Despite the therapeutic potential of targeting non-angiogenic vascularization mechanisms such as VM and VCO, effective clinical interventions have yet to be developed [16, 17].

We hypothesized that inhibiting cancer cell migration is an effective strategy to suppress non-angiogenic vascularization. To explore this hypothesis, we focused on integrins and FAK, which play crucial roles in cancer cell adhesion and migration, as potential targets for the pharmacological inhibition of non-angiogenic vascularization, including VM and VCO. FAK is a cytoplasmic protein tyrosine kinase that drives tumor progression and metastasis by regulating cancer cells and stromal cells within the tumor microenvironment [18, 19]. FAK regulates cell motility, invasion, survival, gene expression, and CSC self-renewal via various intracellular signaling pathways. In addition, FAK is a key downstream signaling molecule of integrin β1 in VM-like vascularization. Integrin β1 knockout suppresses tube formation and reduces FAK Y397 phosphorylation in human fibrosarcoma cell lines [20].

FAK inhibitors have shown promising results in preclinical models, reducing tumor growth and metastasis with minimal side effects [21]. While FAK is recognized as a central regulator of angiogenesis in breast cancer, its potential role in modulating the tumor microenvironment and promoting non-angiogenic vascularization, such as VM and VCO, remains largely unexplored. The precise mechanisms by which FAK inhibition affects non-angiogenic vascularization require further investigation. To our knowledge, this study is the first to examine whether FAK inhibition suppresses cancer cell migration and non-angiogenic vascularization, thereby impeding tumor progression and metastasis.

## Materials and methods

### Cell culture

MDA-MB-231 cells were obtained from the American Type Culture Collection (Manassas, VA, USA), and JIMT-1 cells were obtained from the Deutsche Sammlung von Mikroorganismen und Zellkulturen. We selected JIMT-1 (trastuzumab-resistant, HER2-positive) and MDA-MB-231 (triple-negative) as representative highly malignant breast cancer cell lines to investigate the effects of FAK inhibition [11, 22]. Both cell lines and their derivatives were cultured in DMEM/F12 (Sigma-Aldrich, St Louis, MO, USA) supplemented with 10% FBS (Sigma-Aldrich). Human umbilical vein endothelial cells (HUVECs) were obtained from PromoCell (Heidelberg, Germany) and maintained in Endothelial Cell Growth Medium-2 (PromoCell).

### Western blotting

Cells were cultured on Matrigel (Corning, NY, USA) in Endothelial Basal Medium-2 (EBM-2; Lonza, Basel, Switzerland). The medium was supplemented with Microvascular Endothelial Cell Growth Medium-2 SingleQuots Supplements and growth factors (Lonza), hereafter referred to as complete EBM-2. Cells were treated with complete EBM-2 supplemented with or without defactinib (5 μM for JIMT-1 and 10 µM for MDA-MB-231) for 3 h, collected with Corning Cell Recovery Solution (Corning), lysed in RIPA buffer with protease and phosphatase inhibitors, and analyzed by western blotting (10–30 µg protein), as previously described [23]. The primary and secondary antibodies are listed in Supplementary Table S1.

### Actin fiber, FAK, and integrin β1, and confocal microscopy

JIMT-1 and MDA-MB-231 cells were seeded onto Matrigel-coated four-well chamber slides (Thermo Fisher Scientific, Waltham, MA, USA) and incubated in complete EBM-2 medium with or without defactinib (5 μM for JIMT-1 and 10 µM for MDA-MB-231) for 3 h. After fixation, permeabilization, and blocking, the cells were stained for FAK and integrin β1 (Antibodies shown in Supplementary Table S1). Filamentous actin (F-actin) was visualized using phalloidin-iFluor 594 reagent (Abcam, Cambridge, UK) for 1 h. The nuclei were counterstained with DAPI. Confocal images were captured using an FV3000 confocal microscope (Olympus, Tokyo, Japan). F-actin staining per cell was determined using ImageJ software (National Institutes of Health, Bethesda, MD, USA) and expressed as integrated density. Cell morphology was assessed by measuring roundness, calculated using the following formula:

$${\text{Roundness}}=\frac{4\times \left({\text{area}}/\uppi \right)}{{\left(\text{major axis}\right)}^{2}},$$

where “area” is the surface area of the cell and “major axis” refers to the longest axis of the fitted ellipse.

### Cell migration assay

Cells were seeded into 35-mm μ-Dishes with a two-well culture insert (Ibidi, Martinsried, Germany) and cultured overnight in complete EBM-2 medium. On the following day, the cells were treated with DMSO, 5 μM, or 10 µM defactinib for 3 h. After removing the inserts, phase-contrast images were captured over 48 h using a Leica DMi1 microscope (Leica Microsystems, Wetzlar, Germany) at 5 ×.

### Time-lapse microscopy

Cells precultured in a standard medium were seeded into Matrigel-coated 24-well plates. Cells were cultured in complete EBM-2 medium and treated with 0 µM, 5 µM, or 10 µM defactinib under an IX83 inverted microscope (Olympus) equipped with a temperature-controlled incubator maintained at 37 °C with 5% CO_2_ and 95% air. Phase-contrast images were captured at 2 min and 30-s intervals for up to 24 h after cell seeding.

### Tube formation assay

JIMT-1, MDA-MB-231, and HUVEC cells precultured in a standard medium were seeded into Matrigel-coated plates or µ-Slide 15-well 3D chambered coverslips (Ibidi) in complete EBM-2 medium for up to 72 h. Phase-contrast images were captured using a Leica DMi1 microscope (Leica Microsystems) at 5 ×. The color images were converted to grayscale, resized, and sharpened for clarity using the ImageJ software. Tube formation was quantified by counting the “number of meshes”, an approach similar to that adopted in our former study [23]. To determine the IC_50_, cells were treated with 0, 1, 3, 10, 30, 100, 300, 1000, 3000, and 10,000 nM defactinib. Tube formation was monitored for 72 h using the same imaging and evaluation methods described above.

### MTS assay

JIMT-1 and MDA-MB-231 cells were seeded into 96-well plates, with each well containing complete EBM-2 medium supplemented with 0, 1, 5, and 10 µM defactinib. Cell viability was assessed using the MTS Cell Proliferation Assay Kit (Abcam, ab197010), according to the manufacturer’s protocol.

### Cell transfection with siRNA

JIMT-1 and MDA-MB-231 cells were seeded into 6-well plates (3.0 × 10^6^ cells and 2.5 × 10^6^ cells per well, respectively) in antibiotic-free medium and incubated at 37 °C in 5% CO_2_ for 24 h. Cells were transfected with 25 pmol FAK Silencer siRNAs (S11485, Ambion, Life Technologies, Carlsbad, CA, USA) using Lipofectamine RNAiMAX (Invitrogen, Carlsbad, CA, USA), following the manufacturer’s instructions. Controls were transfected with Silencer Select Negative Control No.1 (Cat. 4,390,843, Ambion, Life Technologies). After 24 h, the medium was replaced with medium supplemented with 10% FBS. On the following day, the cells were passaged into 10-cm dishes. On day 7, the cells were reseeded onto Matrigel-coated plates in complete EBM-2 medium. The cells were monitored for 72 h for tube formation assays or lysed after 3 h for analysis, as previously described.

### Orthotopic tumor models and treatment

Female immunodeficient mice (NOD/SchiJic-scidJcl) purchased from CLEA Japan (Tokyo, Japan) were anesthetized and injected with either 1.2 × 10⁷ MDA-MB-231 or 2.0 × 10⁷ JIMT-1 cells (prewashed in PBS and mixed with 30 µL Matrigel) into the fourth left mammary gland. For each cell line, mice were randomly divided into two groups of 10 individuals: a vehicle group and a defactinib group. Five days after cell injection, the mice were treated orally with 25 mg/kg defactinib (VS-6063) or vehicle, twice daily. Defactinib was diluted in 10% DMSO, 40% PEG300, 5% Tween-80, and 45% saline. Body weight and tumor volume (caliper measurement) were measured every 2 or 3 days. At the study endpoint, the tumors were excised and analyzed. Tumor volumes were calculated using the following formula:

$$V\, = \,\pi /{6}\, \times \,L\, \times \,W\, \times \,H,$$

where *L* is the length, *W* is the width, and *H* is the thickness of the tumor. Half of the excised tumors were cryopreserved (immunofluorescence) and half were formalin-fixed and paraffin-embedded (FFPE).

### H&E staining and immunofluorescence staining of mouse tumors

FFPE mouse tumor sections were stained with H&E to examine the tissue structure, cell distribution, and morphological changes. Tumor proliferation was evaluated via Ki-67 immunostaining using a mouse MIB-1 antibody (Dako) and Histofine Simple Stain MAX PO (M) (Nichirei Bioscience, Tokyo, Japan). Detection was performed with 3,3′-diaminobenzidine (DAB; Wako, Tokyo, Japan), and sections were counterstained with hematoxylin. Ki-67-positive areas were identified on a NIKON ECLIPSE Ci-L plus microscope (NIKON, Tokyo, Japan) at 10 ×. The highest proliferative region (hot spot) was analyzed at 40 ×. The Ki-67 index, calculated as the percentage of positive tumor cells in the hot spot, was independently assessed by two blinded researchers (IS and KK).

For immunofluorescence staining, frozen sections were incubated with the following fluorescently labeled primary antibodies: human serpin family E member 2 (hSERPINE2, Proteintech, Rosemont, IL, USA), mouse platelet endothelial cell adhesion molecule-1 (mPECAM-1, BioLegend, San Diego, CA, USA), and TER119 (BioLegend), a marker for erythroid cells. The nuclei were counterstained with DAPI. Samples were imaged at 40 × using an FV3000 confocal microscope (Olympus). The mPECAM-1-positive vessels with or without TER119-positive cells inside them were quantified. VCO was defined as the presence of vessels in direct contact with or surrounded by a layer of hSERPINE2-positive human breast cancer cells. VCO vessels were also quantified as those with hSERPINE2-positive human breast cancer cells located within a 50 µm radius.

DNA fragmentation was assessed using the TdT-mediated dUTP Nick End Labeling (TUNEL) assay following the manufacturer’s instructions (Merck Millipore, Darmstadt, Germany). The nuclei were counterstained with methyl green. Whole-tumor composite images were captured with a BZ-X810 fluorescence microscope (KEYENCE, Osaka, Japan). Four random fields per tumor were imaged at 10 × using a NIKON ECLIPSE Ci-L plus microscope (NIKON). TUNEL-positive breast cancer cells were quantified and compared between the vehicle and defactinib groups.

### Statistics

Statistical analyses were conducted using GraphPad Prism6 (GraphPad Software, Inc., San Diego, CA, USA) and SPSS software (IBM, Armonk, NY, USA). Student’s *t* tests were used for parametric analyses unless stated otherwise. Mann–Whitney U tests were used for nonparametric analyses. Tumor volumes were compared using multivariate analysis of variance (MANOVA). A *P* value < 0.05 was considered statistically significant. All tests were two-tailed.

## Results

### Defactinib inhibits FAK and its downstream components, affecting the cytoskeleton and cell motility

**Fig. 1:**
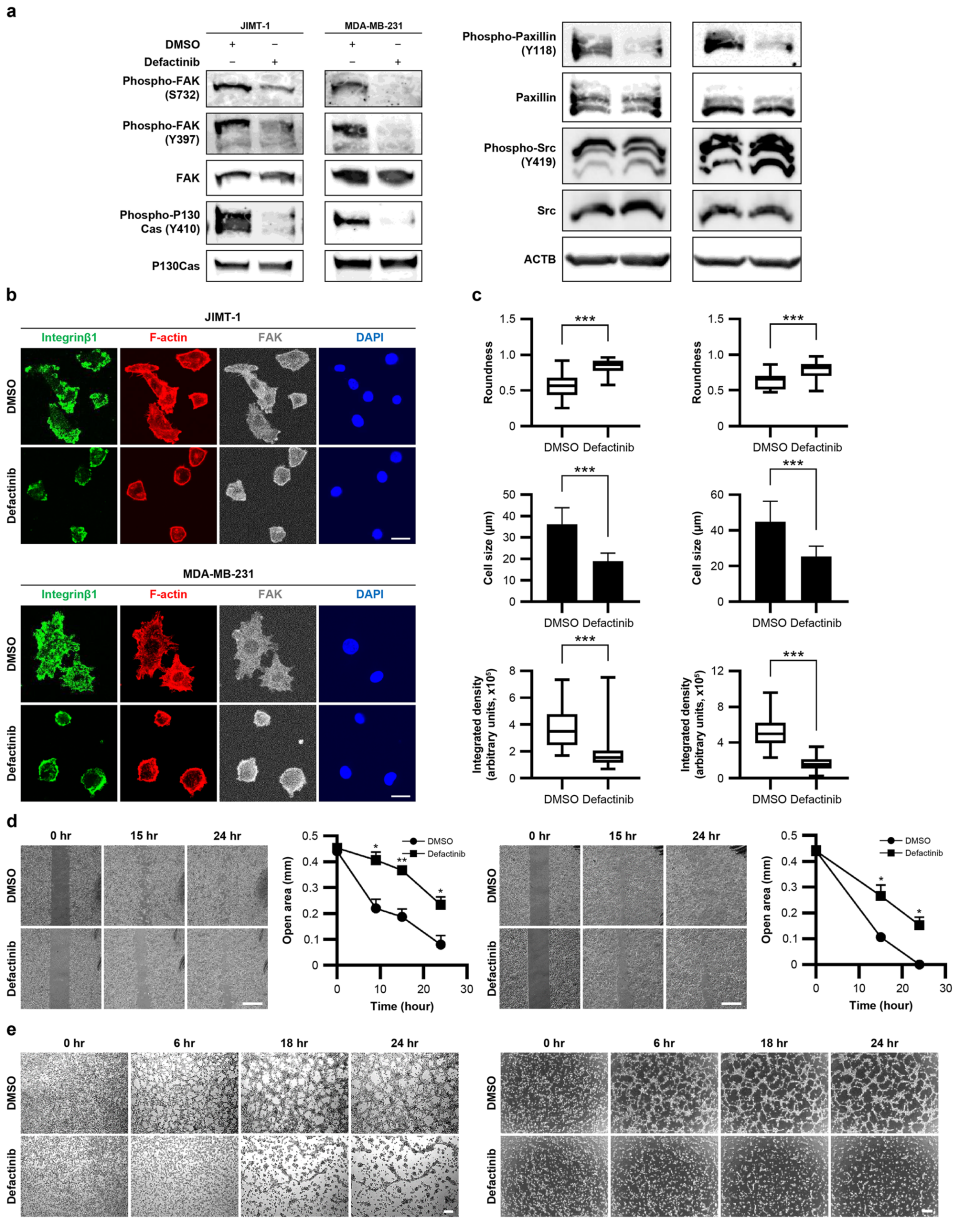
Defactinib inhibits FAK and its downstream components, affecting the cytoskeleton and motility**. a** Western blots of FAK and its downstream components in JIMT-1 and MDA-MB-231 from the tube formation assay.** b** Morphology of cells 3 h after treatment with DMSO or defactinib. Representative confocal microscopy photographs of cells stained with iFluor 594-conjugated phalloidin for F-actin, Alexa Fluor 488 (Mouse IgG secondary antibody) for integrin β1, Alexa Fluor 647 (Rabbit IgG secondary antibody) for FAK, and DAPI. Scale bar, 20 µm.** c** Box plots showing the roundness of cells treated with DMSO or defactinib. JIMT-1 cell treated with DMSO (*n* = 24) or 5 µM defactinib (*n* = 30). MDA-MB-231 cells were treated with DMSO (*n* = 15) or 10 µM defactinib (*n* = 23). The whiskers represent the 10th–90th percentiles. P, Mann–Whitney *U* test. ***P < 0.001. The sizes of cells after treatment with DMSO or defactinib. JIMT-1 cells were treated with DMSO (*n* = 24; mean ± SD) or 5 µM defactinib (*n* = 30*;* mean ± *SD*). MDA-MB-231 cells were treated with DMSO (*n* = 15; mean ± SD) or 10 µM defactinib (*n* = 23; mean ± SD). ***P < 0.001. Box plots of the integrated density showing the amount of F-actin per cell after treatment with DMSO or defactinib. JIMT-1 cells were treated with DMSO (*n* = 24) or 5 µM defactinib (*n* = 30). MDA-MB-231 cells treated with DMSO (*n* = 15) or 10 µM defactinib (*n* = 23). The whiskers show the 10th–90th percentiles. P, Mann–Whitney *U* test. ***P < 0.001. Left: JIMT-1. Right: MDA-MB-231.** d** Cell migration assays after treatment with DMSO or 5 µM defactinib in JIMT-1 cells and 10 µM defactinib in MDA-MB-231 cells (*n* = 3; mean ± SD). Representative photographs displaying the gap width of each cell line. Left: JIMT-1 cells. Right: MDA-MB-231 cells. Scale bar, 0.5 mm. The gap width at 24 h (percentage of gap width at 0, 9, 15, and 24 h) for JIMT-1 cells and at 24 h (percentage of gap width at 0, 15, and 24 h) for MDA-MB-231 cells treated with DMSO or defactinib (*n* = 3; mean ± SD). *P < 0.05; **P < 0.01.** e** Time-lapse microscopy showing tube formation in cells treated with DMSO or 5 µM defactinib for JIMT-1cells and 10 µM defactinib for MDA-MB-231 cells. Left: JIMT-1 cells. Right: MDA-MB-231 cells. Scale bar, 0.2 mm

Phosphorylation of FAK/PTK2 and its downstream effectors, P130Cas and paxillin, was reduced in defactinib-treated JIMT-1 and MDA-MB-231 breast cancer cell lines compared with DMSO-treated cells. However, no reduction in Src expression or phosphorylation was detected (Fig. 1a and Supplementary Fig. S1). Defactinib treatment did not affect FAK localization but inhibited F-actin fiber elongation and lamellipodia formation in JIMT-1 and MDA-MB-231 cells seeded on Matrigel and fixed during the early stages of motility. Defactinib altered the cell morphology. However, the distribution of integrin β1, an upstream regulator of FAK, at the base of the lamellipodia was not changed (Fig. 1b). Defactinib reduced cell size, increased cell roundness, and decreased F-actin content per cell (Fig. 1b and Fig. 1c). Defactinib treatment reduced JIMT-1 and MDA-MB-231 cell motility compared with DMSO-treated cells (Fig. 1d). These findings were verified using time-lapse microscopy of tube formation, showing the inhibition of cell motility and tube formation in defactinib-treated cells (Fig. 1e and Supplementary Videos S1, S2, S3, and S4). Cell viability was slightly decreased at 10 µM defactinib (Supplementary Fig. S2).

### Inhibition of FAK suppresses tube formation in breast cancer cell lines

**Fig. 2:**
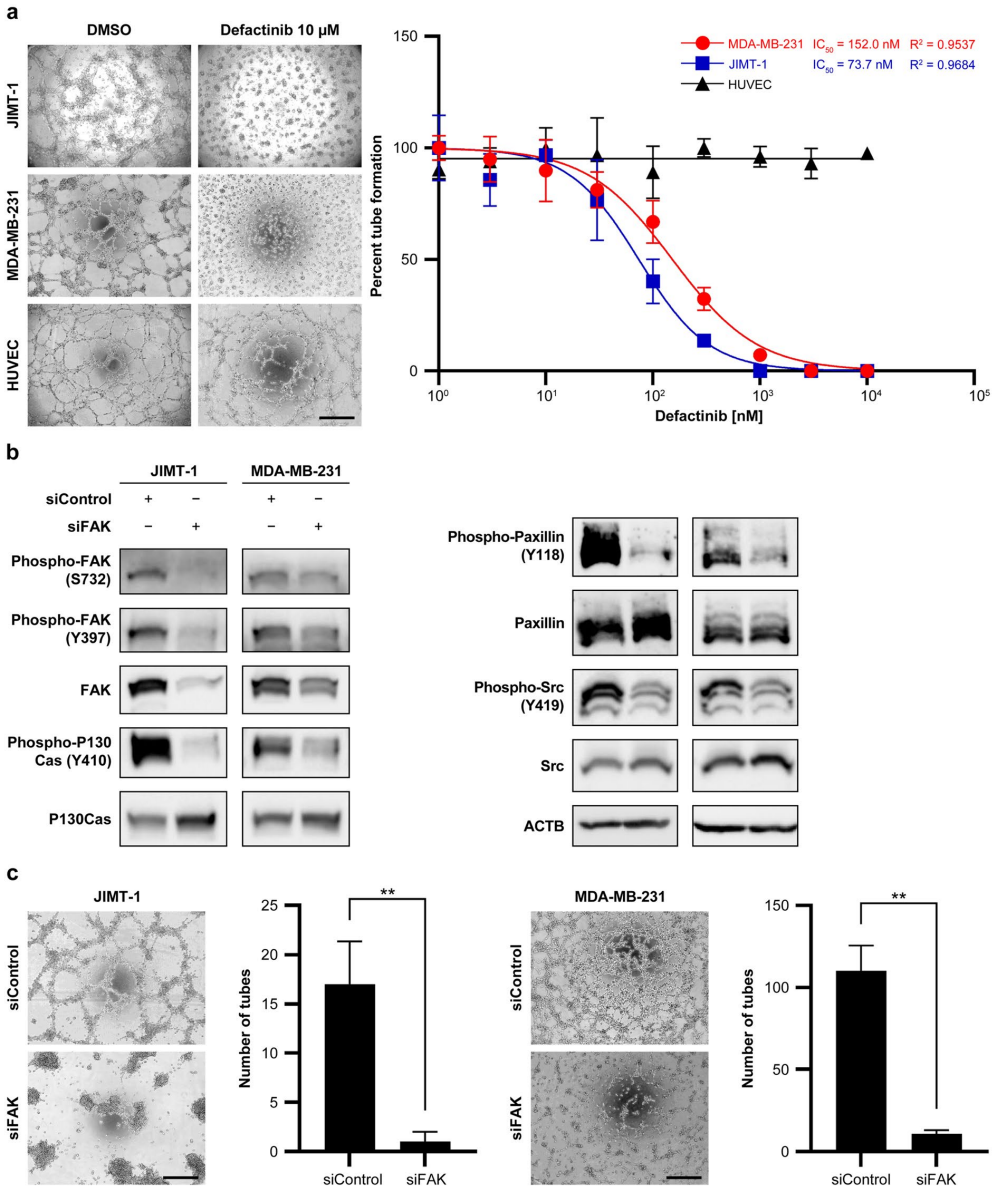
FAK inhibition suppresses tube formation in breast cancer cell lines.** a** Representative photograph showing tube formation in JIMT-1 and MDA-MB-231 cells capable of vasculogenic mimicry and human umbilical vein endothelial cells. Scale bar, 0.5 mm. The concentration-dependent curve of tube formation inhibition using defactinib and IC_50_ values for each of the three cell lines.** b** Western blots for FAK and its downstream components in siFAK-mediated FAK knockdown in JIMT-1 and MDA-MB-231 cells from the tube formation assay.** c** Representative photograph demonstrating tube formation after siFAK-mediated knockdown or negative control in JIMT-1 and MDA-MB-231 cells. Tube formation assay after siFAK-mediated knockdown or negative control in JIMT-1 and MDA-MB-231 cell lines for up to 72 h. The number of tubes formed is shown (*n* = 5; mean ± SD). **P < 0.01. Scale bar, 0.5 mm

Tube formation assays confirmed the VM capability of JIMT-1 and MDA-MB-231 cells. Dose–response studies demonstrated concentration-dependent inhibition of tube formation by defactinib. The IC_50_ values for defactinib-induced inhibition of tube formation were 73.7 nM in JIMT-1 cells and 152.0 nM in MDA-MB-231 cells. Thus, JIMT-1 cells (Fig. 2a) were more sensitive to defactinib than MDA-MB-231 cells. Notably, defactinib did not inhibit tube formation in HUVECs (Fig. 2a). FAK knockdown with siFAK reduced the phosphorylation of FAK/PTK2 and its downstream factors, P130Cas (encoded by *BCAR1*) and paxillin (Fig. 2b and Supplementary Fig. S3). To rule out the potential off-target effects of defactinib, siFAK-mediated FAK knockdown was performed. FAK knockdown also suppressed tube formation in JIMT-1 and MDA-MB-231 cells (Fig. 2c).

### Defactinib inhibits tumor growth in mouse breast cancer models

**Fig. 3:**
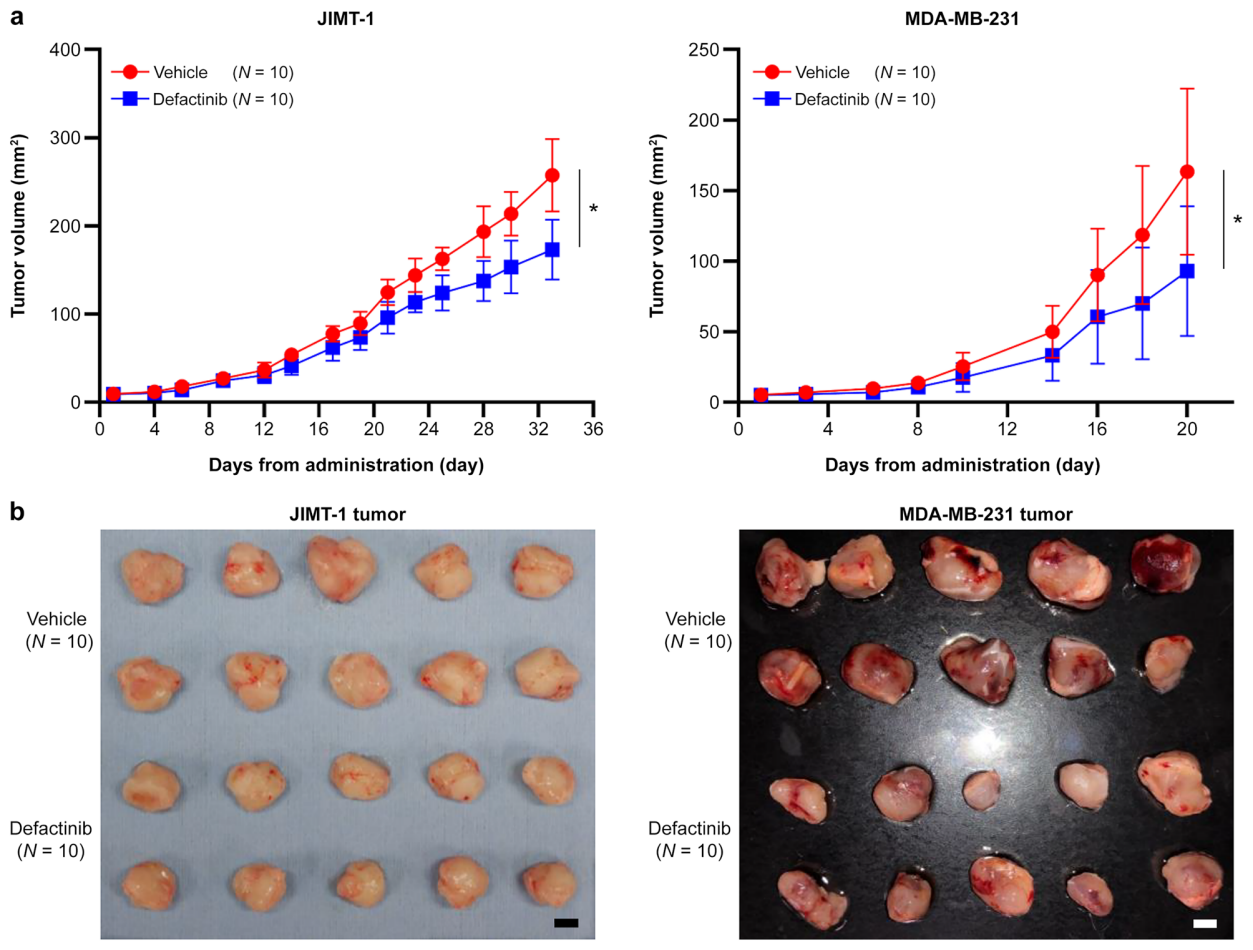
Defactinib inhibits tumor growth in mouse breast cancer models.** a** Tumor growth curves in the orthotopic mouse breast cancer model (NOD/SchiJic-scidJcl) using JIMT-1 or MDA-MB-231 cells from the defactinib-treated (*n* = 10; mean ± SD) or vehicle-treated (*n* = 10; mean ± SD) groups. Data are presented as mean ± SE with 95% confidence intervals. *P* values were calculated by multivariate analysis of variance. *P < 0.05.** b** Photographs of the excised mouse tumors. Scale bar, 1 cm

To investigate the effects of defactinib in vivo, JIMT-1 and MDA-MB-231 cells were transplanted into immunodeficient mice and the tumor sizes and vasculature were evaluated. As shown in Fig. 3a, defactinib treatment significantly suppressed tumor growth in mice transplanted with JIMT-1 or MDA-MB-231 cells compared with vehicle treatment (P < 0.05 for both, MANOVA). No significant weight loss or health issues were observed in any group. Tumors excised from the defactinib-treated mice were smaller and appeared less macroscopically blood-rich (Fig. 3b).

### Defactinib reduces intratumoral vasculature and non-angiogenic vascularization

**Fig. 4:**
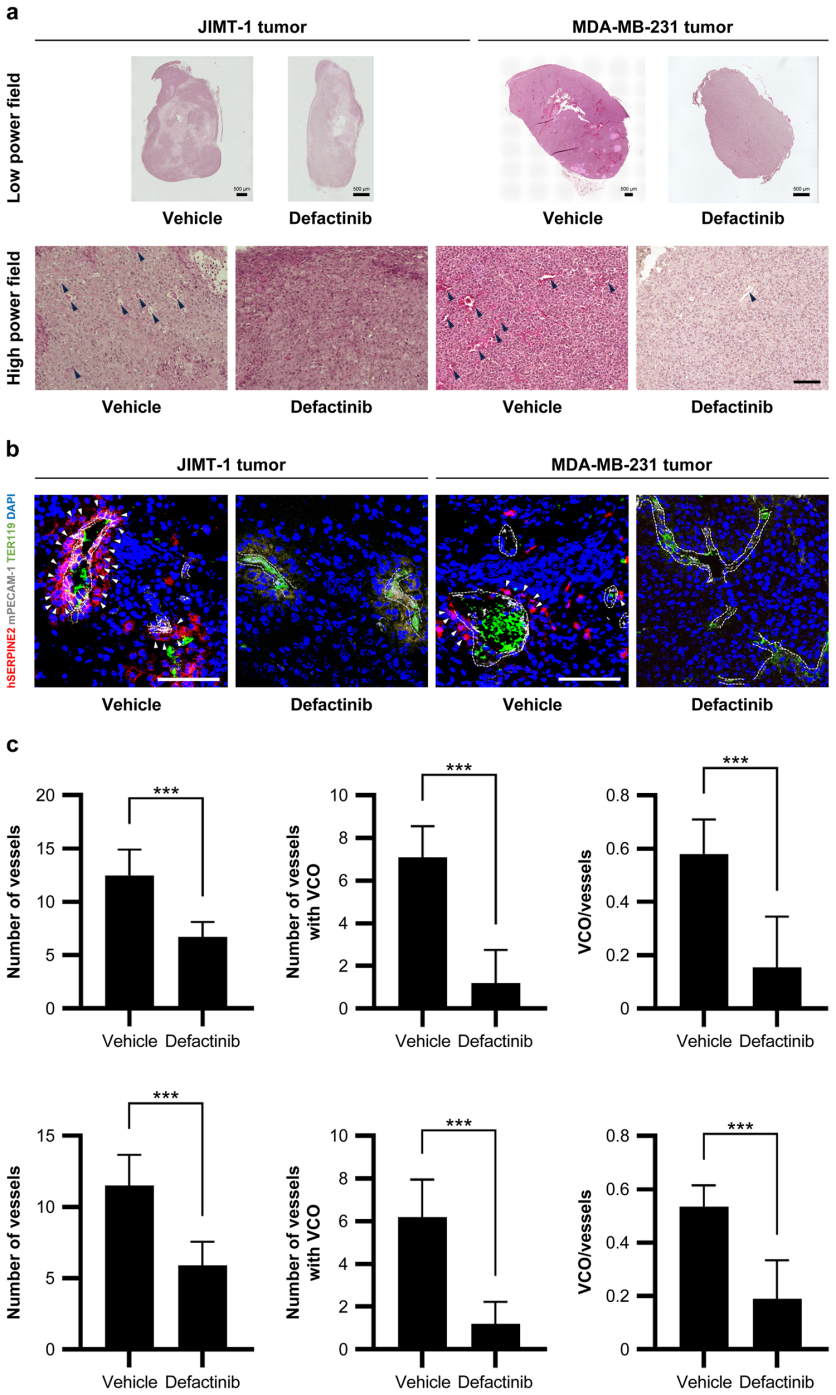
Defactinib reduces intratumoral vasculature and non-angiogenic vascularization**. a** Representative photographs of H&E staining in tumor sections excised from mice treated with vehicle or defactinib (*n* = 10; mean ± SD). The black arrowheads: vascular-like structures within the mouse tumor. Scale bars, 0.5 and 0.1 mm.** b** Representative photographs of the immunofluorescence staining for hSERPINE2 (CoraLite 594; red), mPECAM-1 (Alexa Fluor 647; white), TER119 (Alexa Fluor 488; green), and DAPI (blue) in the tumor frozen sections. Scale bar, 0.1 mm. The area surrounded by a broken line: mouse-derived vessels. White arrowheads: hSERPINE2-positive tumor cells adjacent to mouse endothelial cells.** c** The number of vessels, the number of vessels with VCOs, and their ratios of JIMT-1 and MDA-MB-231 tumors excised from mice treated with vehicle or defactinib (*n* = 10; mean ± SD). The upper row: JIMT-1. The lower row: MDA-MB-231. ***P < 0.001

Tumors in the defactinib-treated group exhibited fewer prominent vascular-like structures—defined as lumens lined by a thin layer of cells, with or without the presence of erythrocytes—compared with those in the control group (Fig. 4a). Multiplex fluorescence immunostaining for hSERPINE2 (a potential indicator of non-angiogenic vascularization), mPECAM-1 (a mouse vascular marker), TER119 (a mouse erythrocyte marker), and DAPI revealed the accumulation of hSERPINE2-positive human breast cancer cells around the mouse-derived vessels, suggesting VCO (Fig. 4b). The number of mouse-derived vessels, VCO-associated (hSERPINE2-positive) mouse-derived vessels, and the ratio of VCO-associated (hSERPINE2-positive) mouse-derived vessels to mouse-derived vessels were significantly reduced in the defactinib-treated groups compared with the vehicle-treated groups (Fig. 4c). VM—defined as TER119-containing channels within the tumor lined by hSERPINE2-positive human breast cancer cells mimicking endothelial structures—was not distinctly observed.

### Defactinib does not significantly increase apoptosis

**Fig. 5:**
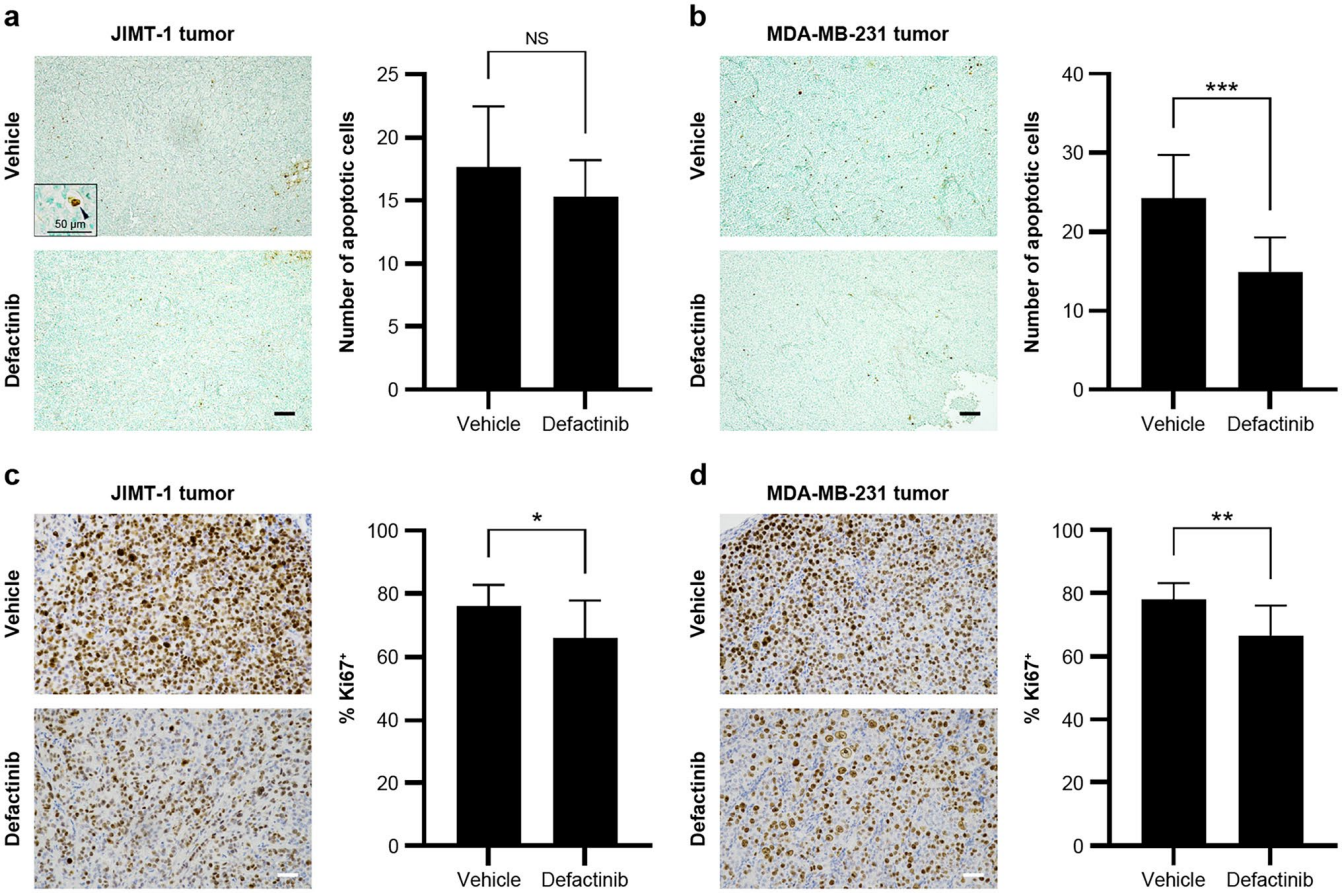
Defactinib does not significantly increase apoptosis. **a, b** Representative photographs of TUNEL staining counterstained with methyl green. Scale bar, 0.1 mm. Inset: a magnified image of a TUNEL-positive apoptotic cancer cell. Scale bar, 50 µm. The number of TUNEL-positive apoptotic cells from tumors excised from mice treated with vehicle or defactinib (*n* = 10; mean ± SD). ns., not significant; ***P < 0.001. **c, d** Representative photographs of the hot spot of immunohistochemical staining for Ki-67 (DAB) counterstained with hematoxylin. Scale bar, 0.1 mm. The Ki-67 positivity index of tumors excised from mice treated with vehicle or defactinib (*n* = 10; mean ± SD). *P < 0.05; **P < 0.01

No significant increases in apoptotic cells were detected after defactinib treatment. Interestingly, MDA-MB-231 tumors in the control group displayed significantly higher levels of apoptosis than the defactinib-treated group (Fig. 5a and b). In contrast, the Ki-67 index within the tumor hot spots was significantly reduced in the defactinib-treated groups compared with the vehicle-treated groups (Fig. 5c and d).

## Discussion

FAK inhibition altered the actin cytoskeleton of cancer cells, resulting in decreased cancer cell motility and tube formation which represents VM capability. The inhibitory effects of defactinib on tube formation was dose-dependent. HUVECs, as normal endothelial cells, form tubes through angiogenesis—a process primarily regulated by the VEGF signaling pathway [24]. Therefore, defactinib had no significant effect on HUVECs. Although defactinib caused a slight decrease in breast cancer cell viability, this effect was modest and did not substantially account for the observed impairment in tube formation. Defactinib treatment suppressed FAK phosphorylation in breast cancer cells, preventing the maturation of focal adhesions. While it did not affect the location of integrin β1 or FAK, it significantly decreased lamellipodia formation and increased cell roundness. Importantly, defactinib treatment limited tumor growth in mouse breast cancer models.

Our findings highlight the role of FAK—a key regulator of cell adhesion and migration—as a potential target to inhibit VM, supported by evidence that FAK knockdown suppressed tube formation. FAK is involved in multiple signaling pathways that promote cancer growth and metastasis. In focal adhesions, the integrin–FAK–actin pathway is a cooperative regulatory mechanism between the cytoskeleton and integrin/FAK-mediated signaling that plays a crucial role in cell motility [25, 26]. Although the precise angiogenic and drug resistance mechanisms are unclear, integrins promote breast cancer invasion and metastasis by regulating integrin β1-FAK signaling and degrading the extracellular matrix [27]. Mechanical stress triggers FAK activation by exposing its active site for Src-mediated phosphorylation, amplifying the signaling pathway [28]. Based on our results, FAK inhibition in breast cancer cells regulates the integrin–FAK–actin pathway at the focal adhesion site to control cell adhesion and migration. The regulation of cell adhesion and migration suppresses cancer progression by inhibiting VM. Notably, Src phosphorylation was reduced only following siRNA-mediated FAK knockdown, but not by defactinib treatment—highlighting FAK's dual role as both a kinase and a scaffolding protein.

VM was not observed in our in vivo mouse model. Instead, we detected VCO, another form of non-angiogenic vascularization. Tumors in defactinib-treated mice appeared less macroscopically blood-rich compared to those in the vehicle-treated group, exhibiting fewer blood vessels and reduced intratumoral hemorrhage. The anti-tumor effects of VCO inhibition were likely due to restricted tumor growth—as indicated by reduced Ki67 expression—rather than increased apoptosis. One possible explanation for the observation of VM in in vitro experiments but not in vivo is that although both VM and VCO are forms of non-angiogenic vascularization, they may not occur simultaneously. VCO is more commonly observed in early stage tumors or metastatic sites [29], while VM typically arises under specific conditions, such as hypoxia, cellular stress, or later stages of tumor progression [30, 31].

Integrins, especially integrin β1, and Src may contribute to non-angiogenic vascularization. Therefore, it is essential to investigate whether FAK represents the most effective therapeutic target [20]. Integrin β1 is known to contribute to antiangiogenic therapy resistance, such as in bevacizumab-resistant glioblastoma [32]. However, integrins play complex and often redundant roles not only in cancer biology, but also in the maintenance of normal tissue homeostasis [33], complicating efforts to target them therapeutically. To date, no clinical trials have demonstrated the efficacy of integrin inhibitors [34]. Similarly, the efficacy of Src inhibitors in targeting non-angiogenic vascularization remains unclear, with no significant clinical success reported [35, 36]. Given the results of our study, along with evidence that defactinib has shown a tolerable safety profile in Phase Ⅰ clinical trials [37, 38], defactinib could be a viable candidate for clinical application.

Determining if FAK inhibitors should be administered alone or in combination with other medications in a clinical setting is crucial. Given the heterogeneity of breast cancer, targeting FAK alone may not be sufficient. Preclinical studies suggest that FAK inhibition lowers CSC activity and self-renewal potential. Combining FAK inhibitors with adjuvant treatments (lapatinib or paclitaxel) further reduces CSC activity in HER2-positive and TNBC cell lines, presenting a promising treatment strategy for high-risk patients [39, 40]. Defactinib may be an effective treatment option for patients with breast cancer who are resistant to existing antiangiogenic medicines, and combination therapy with bevacizumab may be possible.

Several limitations of this study should be considered. First, while VM is commonly assessed using the in vitro tube formation assay, there is currently no well-established method for evaluating non-angiogenic vascularization. Thus, determining the effects of defactinib on non-angiogenic vascularization such as VM and VCO is challenging. Despite the lack of standardized markers or evaluation procedures, we believe that our experimental results provide strong evidence that tumor growth suppression was achieved by inhibiting non-angiogenic vascularization. Future research should focus on developing more reliable in vitro methods for assessing both VM and VCO, as well as in vivo models incorporating intravital imaging to quantify intratumoral blood flow. Further studies are needed to determine the conditions under which VM, in addition to VCO, can be observed in vivo. Additionally, the potential effects of defactinib on angiogenesis should be assessed.

In conclusion, high-grade breast cancers, such as trastuzumab-resistant HER2-positive breast cancer and TNBC, exhibit rapid tumor growth and spread due to increased blood flow acquisition via non-angiogenic vascularization. Existing drug therapies are not sufficiently effective; hence, the development of novel strategies targeting VM and VCO is needed. Our findings demonstrate that FAK inhibition reduces cancer cell motility and tube formation in vitro, and suppresses tumor growth in mouse models by inhibiting non-angiogenic vascularization. Defactinib may be a promising treatment for patients with breast cancer who are resistant to current antiangiogenic medicines. For practical clinical application, further research is required.

## Supplementary Information

Below is the link to the electronic supplementary material.

Supplementary file1 (DOCX 375 KB)

Supplementary file2 (TIF 965 KB)

Supplementary file3 (TIF 429 KB)

Supplementary file4 (TIF 809 KB)

Supplementary file5 (AVI 7384 KB)

Supplementary file6 (AVI 9323 KB)

Supplementary file7 (AVI 4466 KB)

Supplementary file8 (AVI 3577 KB)

## Data Availability

The data generated in this study are available upon request from the corresponding author.

## References

[1] Goldfarb SB, Hudis C, Dickler MN. Bevacizumab in metastatic breast cancer: when may it be used? Ther Adv Med Oncol. 2011;3:85–93. 21789158 10.1177/1758834010397627 PMC3126041

[2] Andre F, Deluche E, Bonnefoi H. Bevacizumab: the phoenix of breast oncology? Lancet Oncol. 2015;16:600–1. 25975636 10.1016/S1470-2045(15)70201-9

[3] Ueda S, Saeki T, Osaki A, Yamane T, Kuji I. Bevacizumab induces acute hypoxia and cancer progression in patients with refractory breast cancer: multimodal functional imaging and multiplex cytokine analysis. Clin Cancer Res. 2017;23:5769–78. 28679773 10.1158/1078-0432.CCR-17-0874

[4] Xu J, Yang X, Deng Q, Yang C, Wang D, Jiang G, et al. TEM8 marks neovasculogenic tumor-initiating cells in triple-negative breast cancer. Nat Commun. 2021;12:4413. 34285210 10.1038/s41467-021-24703-7 PMC8292527

[5] Hendrix MJC, Seftor EA, Hess AR, Seftor REB. Vasculogenic mimicry and tumour-cell plasticity: lessons from melanoma. Nat Rev Cancer. 2003;3:411–21. 12778131 10.1038/nrc1092

[6] Wagenblast E, Soto M, Gutiérrez-Ángel S, Hartl CA, Gable AL, Maceli AR, et al. A model of breast cancer heterogeneity reveals vascular mimicry as a driver of metastasis. Nature. 2015;520:358–62. 25855289 10.1038/nature14403 PMC4634366

[7] Maniotis AJ, Folberg R, Hess A, Seftor EA, Gardner LMG, Pe’er J, et al. Vascular channel formation by human melanoma cells in vivo and in vitro: vasculogenic mimicry. Am J Pathol. 1999;155:739–52. 10487832 10.1016/S0002-9440(10)65173-5 PMC1866899

[8] Cao Z, Bao M, Miele L, Sarkar FH, Wang Z, Zhou Q. Tumour vasculogenic mimicry is associated with poor prognosis of human cancer patients: a systemic review and meta-analysis. Eur J Cancer. 2013;49:3914–23. 23992642 10.1016/j.ejca.2013.07.148

[9] Luo Q, Wang J, Zhao W, Peng Z, Liu X, Li B, et al. Vasculogenic mimicry in carcinogenesis and clinical applications. J Hematol Oncol. 2020;13:19. 32169087 10.1186/s13045-020-00858-6 PMC7071697

[10] Gupta PB, Onder TT, Jiang G, Tao K, Kuperwasser C, Weinberg RA, et al. Identification of selective inhibitors of cancer stem cells by high-throughput screening. Cell. 2009;138:645–59. 19682730 10.1016/j.cell.2009.06.034 PMC4892125

[11] Hori A, Shimoda M, Naoi Y, Kagara N, Tanei T, Miyake T, et al. Vasculogenic mimicry is associated with trastuzumab resistance of HER2-positive breast cancer. Breast Cancer Res. 2019;21:88. 31387614 10.1186/s13058-019-1167-3 PMC6683360

[12] Angara K, Borin TF, Arbab AS. Vascular mimicry: a novel neovascularization mechanism driving antiangiogenic therapy (AAT) resistance in glioblastoma. Transl Oncol. 2017;10:650–60. 28668763 10.1016/j.tranon.2017.04.007 PMC5496207

[13] Kirschmann DA, Seftor EA, Hardy KM, Seftor REB, Hendrix MJC. Molecular pathways: vasculogenic mimicry in tumor cells: diagnostic and therapeutic implications. Clin Cancer Res. 2012;18:2726–32. 22474319 10.1158/1078-0432.CCR-11-3237 PMC3354024

[14] Kuczynski EA, Vermeulen PB, Pezzella F, Kerbel RS, Reynolds AR. Vessel co-option in cancer. Nat Rev Clin Oncol. 2019;16:469–93. 30816337 10.1038/s41571-019-0181-9

[15] Liu Z-L, Chen H-H, Zheng L-L, Sun L-P, Shi L. Angiogenic signaling pathways and antiangiogenic therapy for cancer. Sig Transduct Target Ther. 2023;8:198. 10.1038/s41392-023-01460-1 PMC10175505 37169756

[16] Kuczynski EA, Reynolds AR. Vessel co-option and resistance to antiangiogenic therapy. Angiogenesis. 2020;23:55–74. 31865479 10.1007/s10456-019-09698-6

[17] Frentzas S, Simoneau E, Bridgeman VL, Vermeulen PB, Foo S, Kostaras E, et al. Vessel co-option mediates resistance to antiangiogenic therapy in liver metastases. Nat Med. 2016;22:1294–302. 27748747 10.1038/nm.4197 PMC5104270

[18] Jean C, Chen XL, Nam J-O, Tancioni I, Uryu S, Lawson C, et al. Inhibition of endothelial FAK activity prevents tumor metastasis by enhancing barrier function. J Cell Biol. 2014;204:247–63. 24446483 10.1083/jcb.201307067 PMC3897185

[19] Miyazaki T, Kato H, Nakajima M, Sohda M, Fukai Y, Masuda N, et al. FAK overexpression is correlated with tumour invasiveness and lymph node metastasis in oesophageal squamous cell carcinoma. Br J Cancer. 2003;89:140–5. 12838315 10.1038/sj.bjc.6601050 PMC2394235

[20] Kawahara R, Niwa Y, Simizu S. Integrin β1 is an essential factor in vasculogenic mimicry of human cancer cells. Cancer Sci. 2018;109:2490–6. 29900640 10.1111/cas.13693 PMC6113431

[21] Sulzmaier FJ, Jean C, Schlaepfer DD. FAK in cancer: mechanistic findings and clinical applications. Nat Rev Cancer. 2014;14:598–610. 25098269 10.1038/nrc3792 PMC4365862

[22] Tanner M, Kapanen AI, Junttila T, Raheem O, Grenman S, Elo J, et al. Characterization of a novel cell line established from a patient with Herceptin-resistant breast cancer. Mol Cancer Ther. 2004;3:1585–92. 15634652

[23] Chihara Y, Shimoda M, Hori A, Ohara A, Naoi Y, Ikeda J, et al. A small-molecule inhibitor of SMAD3 attenuates resistance to anti-HER2 drugs in HER2-positive breast cancer cells. Breast Cancer Res Treat. 2017;166:55–68. 28702892 10.1007/s10549-017-4382-6

[24] Lamalice L, Le Boeuf F, Huot J. Endothelial cell migration during angiogenesis. Circ Res. 2007;100:782–94. 17395884 10.1161/01.RES.0000259593.07661.1e

[25] Mierke CT, Fischer T, Puder S, Kunschmann T, Soetje B, Ziegler WH. Focal adhesion kinase activity is required for actomyosin contractility-based invasion of cells into dense 3D matrices. Sci Rep. 2017;7:42780. 28202937 10.1038/srep42780 PMC5311912

[26] Yu H, Gao M, Ma Y, Wang L, Shen Y, Liu X. Inhibition of cell migration by focal adhesion kinase: time-dependent difference in integrin-induced signaling between endothelial and hepatoblastoma cells. Int J Mol Med. 2018;41:2573–82. 29484384 10.3892/ijmm.2018.3512 PMC5846670

[27] Yousefi H, Vatanmakanian M, Mahdiannasser M, Mashouri L, Alahari NV, Monjezi MR, et al. Understanding the role of integrins in breast cancer invasion, metastasis, angiogenesis, and drug resistance. Oncogene. 2021;40:1043–62. 33420366 10.1038/s41388-020-01588-2

[28] Bauer MS, Baumann F, Daday C, Redondo P, Durner E, Jobst MA, et al. Structural and mechanistic insights into mechanoactivation of focal adhesion kinase. Proc Natl Acad Sci USA. 2019;116:6766–74. 30877242 10.1073/pnas.1820567116 PMC6452671

[29] Donnem T, Hu J, Ferguson M, Adighibe O, Snell C, Harris AL, et al. Vessel co-option in primary human tumors and metastases: an obstacle to effective antiangiogenic treatment? Cancer Med. 2013;2:427–36. 24156015 10.1002/cam4.105 PMC3799277

[30] Qiao L, Liang N, Zhang J, Xie J, Liu F, Xu D, et al. Advanced research on vasculogenic mimicry in cancer. J Cell Mol Med. 2015;19:315–26. 25598425 10.1111/jcmm.12496 PMC4407602

[31] Tang H, Chen L, Liu X, Zeng S, Tan H, Chen G. Pan-cancer dissection of vasculogenic mimicry characteristic to provide potential therapeutic targets. Front Pharmacol. 2024;15:1346719. 38694917 10.3389/fphar.2024.1346719 PMC11061449

[32] Carbonell WS, DeLay M, Jahangiri A, Park CC, Aghi MK. β1 integrin targeting potentiates antiangiogenic therapy and inhibits the growth of bevacizumab-resistant glioblastoma. Cancer Res. 2013;73:3145–54. 23644530 10.1158/0008-5472.CAN-13-0011 PMC4040366

[33] Leask A. Integrin β1: a mechanosignaling sensor essential for connective tissue deposition by fibroblasts. Adv Wound Care. 2013;2:160–6. 10.1089/wound.2012.0365 PMC3840546 24527339

[34] Bell-McGuinn KM, Matthews CM, Ho SN, Barve M, Gilbert L, Penson RT, et al. A phase Ⅱ, single-arm study of the anti-α5β1 integrin antibody volociximab as monotherapy in patients with platinum-resistant advanced epithelial ovarian or primary peritoneal cancer. Gynecol Oncol. 2011;121:273–9. 21276608 10.1016/j.ygyno.2010.12.362 PMC4426879

[35] Eom K-Y, Cho BJ, Choi EJ, Kim J-H, Chie EK, Wu H-G, et al. The effect of chemoradiotherapy with SRC tyrosine kinase inhibitor, PP2 and temozolomide on malignant glioma cells in vitro and in vivo. Cancer Res Treat. 2016;48:687–97. 26044161 10.4143/crt.2014.320 PMC4843743

[36] Gucalp A, Sparano JA, Caravelli J, Santamauro J, Patil S, Abbruzzi A, et al. Phase II trial of saracatinib (AZD0530), an oral SRC-inhibitor for the treatment of patients with hormone receptor-negative metastatic breast cancer. Clin Breast Cancer. 2011;11:306–11. 21729667 10.1016/j.clbc.2011.03.021 PMC3222913

[37] Jones SF, Siu LL, Bendell JC, Cleary JM, Razak ARA, Infante JR, et al. A phase I study of VS-6063, a second-generation focal adhesion kinase inhibitor, in patients with advanced solid tumors. Invest New Drugs. 2015;33:1100–7. 26334219 10.1007/s10637-015-0282-y

[38] Shimizu T, Fukuoka K, Takeda M, Iwasa T, Yoshida T, Horobin J, et al. A first-in-Asian phase 1 study to evaluate safety, pharmacokinetics and clinical activity of VS-6063, a focal adhesion kinase (FAK) inhibitor in Japanese patients with advanced solid tumors. Cancer Chemother Pharmacol. 2016;77:997–1003. 27025608 10.1007/s00280-016-3010-1 PMC4844649

[39] Timbrell S, Aglan H, Cramer A, Foden P, Weaver D, Pachter J, et al. Fak inhibition alone or in combination with adjuvant therapies reduces cancer stem cell activity. NPJ Breast Cancer. 2021;7:65. 34050172 10.1038/s41523-021-00263-3 PMC8163772

[40] Dawson JC, Serrels A, Stupack DG, Schlaepfer DD, Frame MC. Targeting FAK in anticancer combination therapies. Nat Rev Cancer. 2021;21:313–24. 33731845 10.1038/s41568-021-00340-6 PMC8276817

