# Supplementary material for: Targeting focal adhesion kinase inhibits cell migration and non-angiogenic vascularization in malignant breast cancer
Source: Breast Cancer. 2025 Oct 28;33(1):188–99. doi: 10.1007/s12282-025-01792-6 (PMC12789117; doi:10.1007/s12282-025-01792-6)
Supplement: Supplementary file 1 — Supplementary file1 (DOCX 375 KB) [file 12282_2025_1792_MOESM1_ESM.docx]

**Original Article**

**Targeting focal adhesion kinase inhibits cell migration and non-angiogenic vascularization in malignant breast cancer**

Misato Masuyama, Masafumi Shimoda, Ikumi Seto, Kaori Kikumori, Kaori Abe, Nanae Masunaga, Chieko Mishima, Masami Tsukabe, Tetsuhiro Yoshinami, Yoshiaki Sota, Tomohiro Miyake, Tomonori Tanei, and Kenzo Shimazu

Department of Breast and Endocrine Surgery, Graduate School of Medicine, The University of Osaka

Corresponding author: Masafumi Shimoda

Department of Breast and Endocrine Surgery, The University of Osaka Graduate School of Medicine

2-2-E10, Yamadaoka, Suita, Osaka, Japan, 565-0871

Telephone: 06-6879-3772

Fax: 06-6879-3779, [mshimoda@onsurg.med.osaka-u.ac.jp](mailto:mshimoda@onsurg.med.osaka-u.ac.jp)

ORCID ID: 0000-0002-0832-9877

**Supplementary Information**

**Supplementary Figure 1** Western blots of FAK and its downstream components in JIMT-1 and MDA-MB-231 cells from the tube formation assay.





**Supplementary Figure 2** Cell viability of JIMT-1 and MDA-MB-231 cells treated by defactinib from the MTS assay (*n* = 6; mean ± SD). **P* < 0.05.





**Supplementary Figure 3** Western blots of FAK and its downstream components in siFAK-mediated FAK knockdown cells of JIMT-1 and MDA-MB-231 from the tube formation assay.





**Supplementary Table 1** Antibodies used for western blotting

| Antigen | Company | Clone | Catalog no. |
| --- | --- | --- | --- |
| Primary antibodies for western blotting | | | |
| p-FAK (S732) | Abcam |  | Ab4792 |
| p-FAK (Y397) | Invitrogen |  | 44-624 G |
| FAK | Cell Signaling Technology | D507U | 71433 |
| p-P130Cas (Y410) | Cell Signaling Technology |  | 4011 |
| P130Cas | Cell Signaling Technology | E1L9H | 13846 |
| p-Paxillin (Y118) | Cell Signaling Technology | E909F | 69363 |
| Paxillin | Cell Signaling Technology |  | 2542 |
| p-Src (Y419) | Cell Signaling Technology | D49G4 | 6943 |
| Src | Cell Signaling Technology | 36D10 | 2109 |
| ACTB | Cell Signaling Technology | D6A8 | 12620 |
| Secondary antibodies for western blotting | | | |
| Mouse IgG | Cell Signaling Technology |  | 7076 |
| Rabbit IgG | Cell Signaling Technology |  | 7074 |
| Primary antibodies for immunohistochemistry | | | |
| Integrinβ1 | Abcam | 12G10 | Ab30394 |
| FAK | Cell Signaling Technology | D507U | 71433 |
| hSERPINE2  (CoraLite 594) | Proteintech | 1E11F12 | CL594-66203 |
| mPECAM-1  (Alexa Fluor 647) | BioLegend | MEC13.3 | 102515 |
| TER119  (Alexa Fluor 488) | BioLegend | TER-119 | 116215 |
| Ki-67 | Dako | MIB-1 | IR62661-2 |
| Secondary antibodies for immunohistochemistry | | | |
| Mouse IgG  (Alexa Fluor 488) | Invitrogen |  | A11017 |
| Rabbit IgG  (Alexa Fluor 647) | Invitrogen |  | A48285 |

**Supplementary Video 1** Time-lapse video demonstrating a tube formation assay using JIMT-1 cells treated with DMSO. (AVI 7.20 MB)

**Supplementary Video 2** Time-lapse video demonstrating a tube formation assay using JIMT-1 cells treated with 5 µM defactinib. (AVI 9.10 MB)

**Supplementary Video 3** Time-lapse video demonstrating a tube formation assay using MDA-MB-231 cells treated with DMSO. (AVI 4.36 MB)

**Supplementary Video 4** Time-lapse video demonstrating a tube formation assay using MDA-MB-231 cells treated with 10 µM defactinib. (AVI 3.49 MB)
