# Supplementary figures and images for: Targeting focal adhesion kinase inhibits cell migration and non-angiogenic vascularization in malignant breast cancer
Source: Breast Cancer. 2025 Oct 28;33(1):188–99. doi: 10.1007/s12282-025-01792-6 (PMC12789117; doi:10.1007/s12282-025-01792-6)

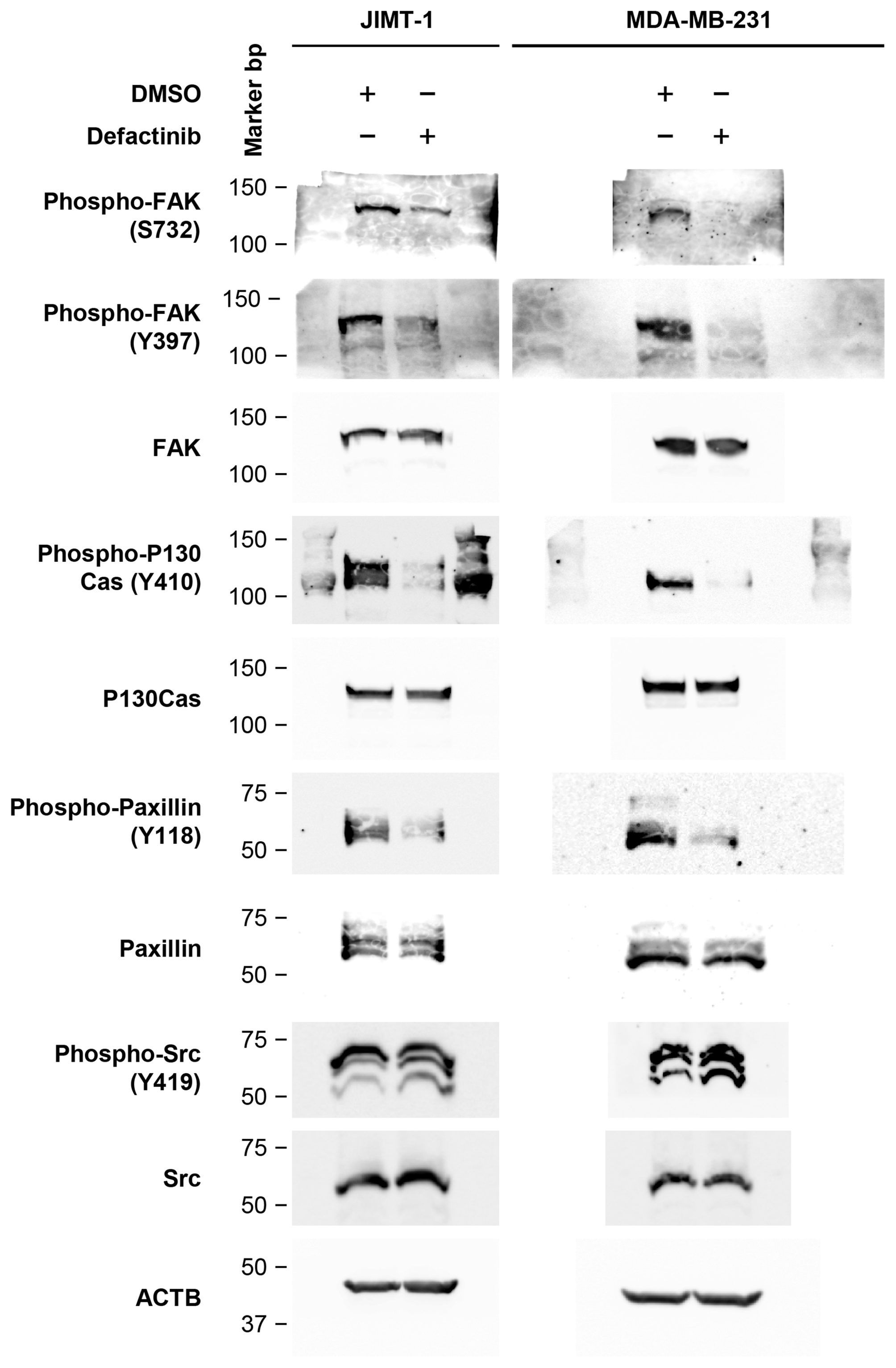

Supplement: Supplementary file 2 — Supplementary file2 (TIF 965 KB) [file 12282_2025_1792_MOESM2_ESM.tif]

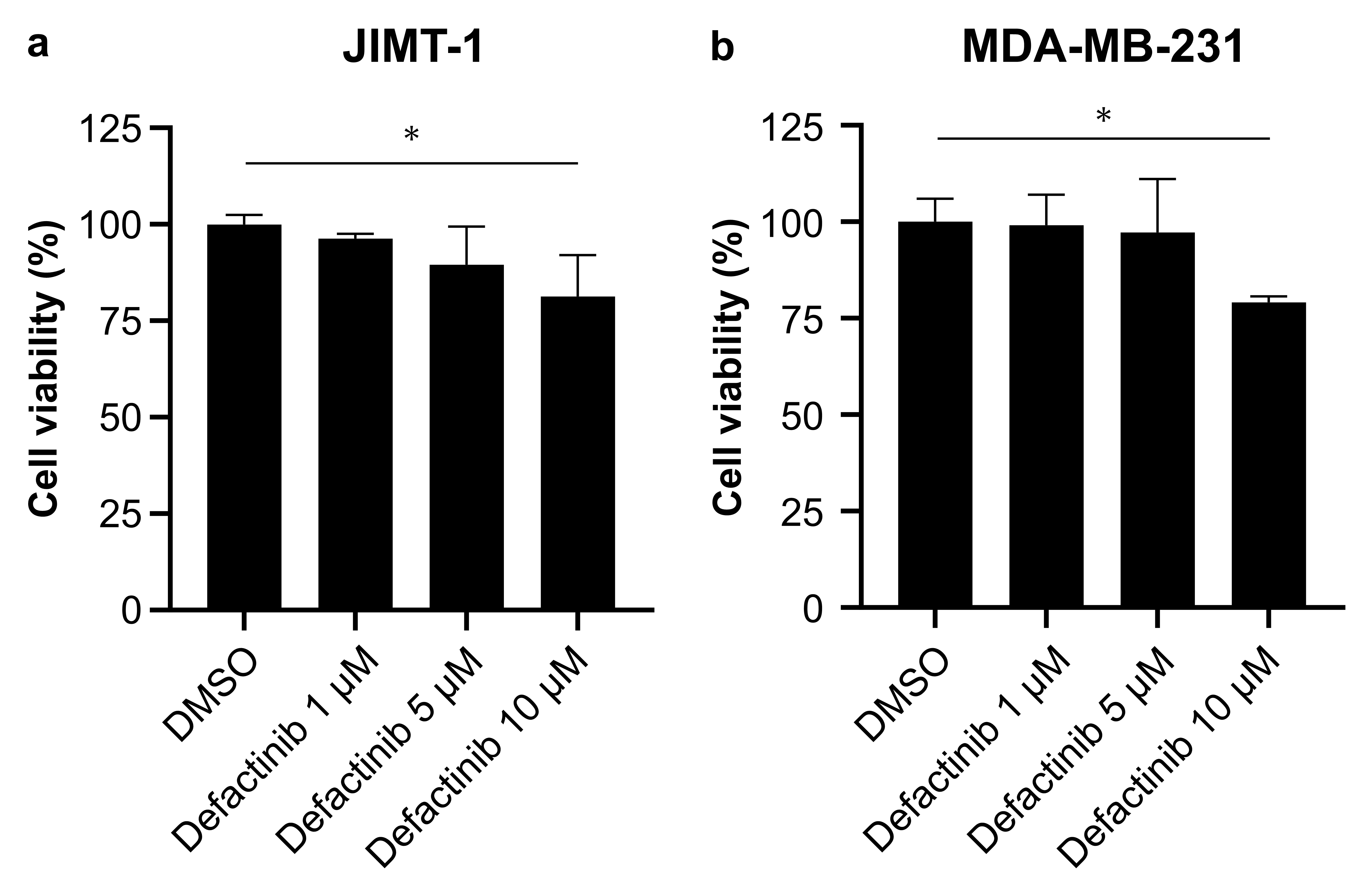

Supplement: Supplementary file 3 — Supplementary file3 (TIF 429 KB) [file 12282_2025_1792_MOESM3_ESM.tif]

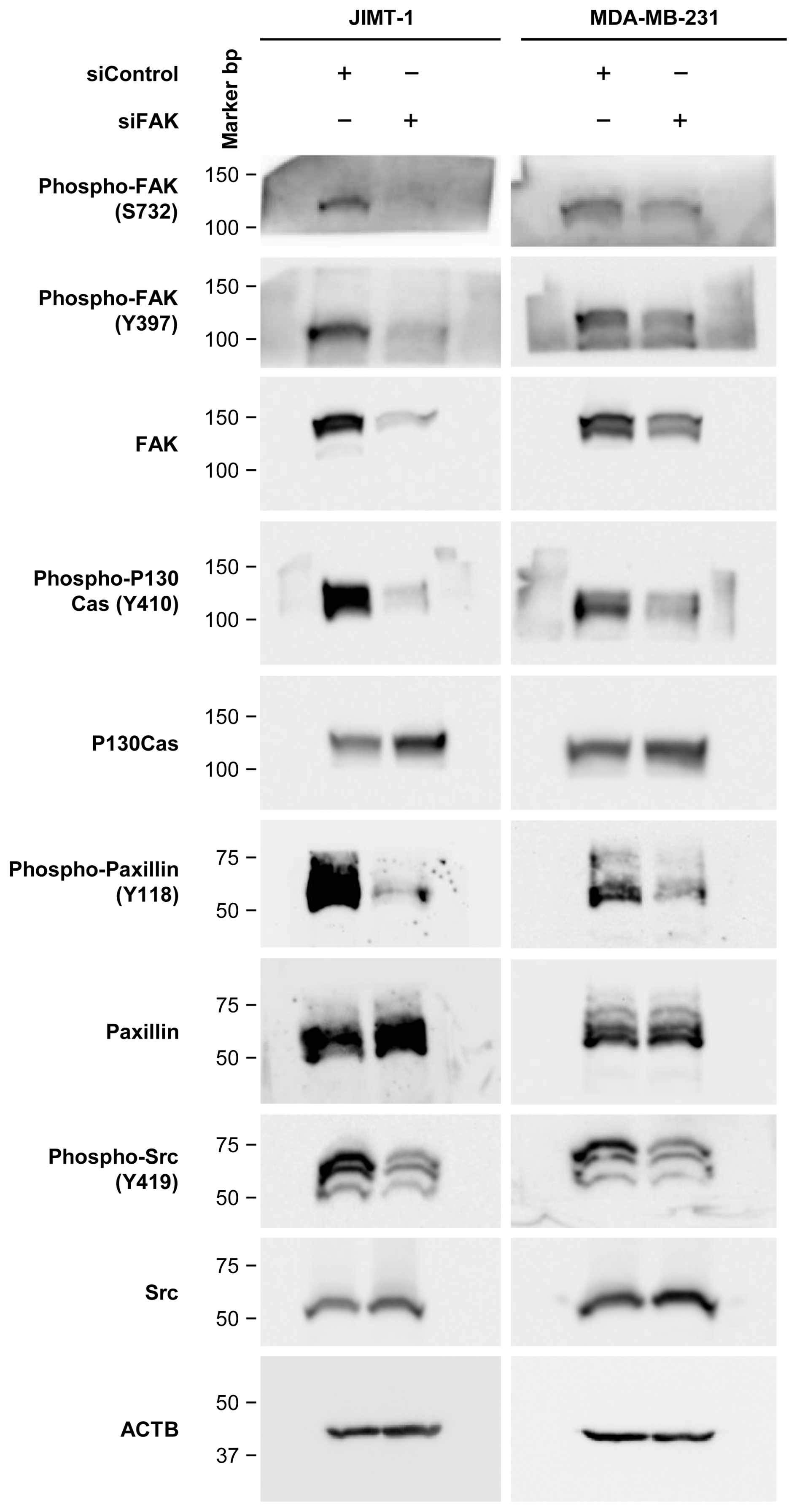

Supplement: Supplementary file 4 — Supplementary file4 (TIF 809 KB) [file 12282_2025_1792_MOESM4_ESM.tif]
